# Quantification of the Retention and Disassembly of Virus Particles by a PEI-Functionalized Microfiltration Membrane

**DOI:** 10.1021/acsapm.2c00560

**Published:** 2022-06-27

**Authors:** Swarupa Chatterjee, Robert Molenaar, Wiebe M. de Vos, Hendrik D. W. Roesink, R. Martijn Wagterveld, Jeroen J. L. M. Cornelissen, Mireille M. A. E. Claessens, Christian Blum

**Affiliations:** † Nanobiophysics (NBP), MESA + Institute for Nanotechnology and Technical Medical Centre, Faculty of Science and Technology, University of Twente, PO Box 217, 7500 AE Enschede, The Netherlands; ‡ Wetsus, European Centre of Excellence for Sustainable Water Technology, Oostergoweg 9, 8911 MA Leeuwarden, The Netherlands; § Membrane Science & Technology cluster (MST), MESA+ Institute for Nanotechnology, Faculty of Science and Technology, University of Twente, PO Box 217, 7500 AE Enschede, The Netherlands; ∥ Biomolecular Nanotechnology (BNT), MESA + Institute for Nanotechnology, Faculty of Science and Technology, University of Twente, PO Box 217, 7500 AE Enschede, The Netherlands

**Keywords:** virucidal surface, fluorescence microscopy, fluorescence spectroscopy, microfiltration, virus inactivation, virus retention

## Abstract

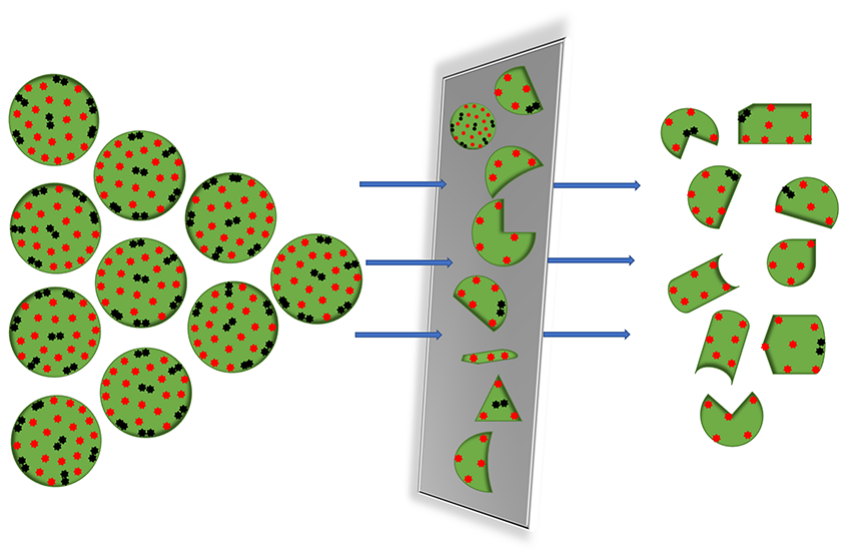

Monitoring the performance of polymer-functionalized surfaces that aim at removing and inactivating viruses is typically labor-intensive and time-consuming. This hampers the development and optimization of such surfaces. Here we present experiments of low complexity that can be used to characterize and quantify the antiviral properties of polymer-functionalized surfaces. We showcase our approach on polyethylenimine (PEI)-coated poly(ether sulfone) (PES) microfiltration membranes. We use a fluorescently labeled model virus to quantify both virus removal and inactivation. We directly quantify the log removal of intact viruses by this membrane using single particle counting. Additionally, we exploit the change in photophysical properties upon disassembly of the virus to show that viruses are inactivated by the PEI coating. Although only a small fraction of intact viruses can pass the membrane, a considerable fraction of inactivated, disassembled viruses are found in the filtrate. Fluorescence microscopy experiments show that most of the viruses left behind on the microfiltration membrane are in the inactivated, disassembled state. Combined, our fluorescence microscopy and spectroscopy experiments show that not only does the model virus adsorb to the PEI coating on the membrane but also the interaction with PEI results in the disassembly of the virus capsid.

## Introduction

A large field in virus research addresses the development of methods for the inactivation and removal of viruses in, for example, water and biological fluids. The most straightforward way to remove viruses is based on their size, for example, by ultrafiltration membranes that are used for water filtration.^1^ A different approach relies on (polymer)-functionalized surfaces that interact with the virus particles.^2^ These interactions may result in removal by virus adsorption on the surface and/or in inactivation by the surface. One example of functionalized surfaces for virus removal and inactivation is the polymer-functionalized microfiltration membrane for gravity or low pressure driven water filtration. Nonfunctionalized microfiltration membranes poorly retain viruses as their large pore size allows for the relatively unhindered passage of viruses. To make microfiltration membranes suitable for virus removal, functionalization of the membrane is required. Membrane functionalizations that have been realized, on a lab scale, to decrease the virus load in water include coating with the cationic polymer polyethylenimine (PEI) and grafting with zwitterionic polymer hydrogels.^3,4^ These functionalized membranes have been shown to effectively remove infective virus particles. However, the mechanism behind the reduction of infectious particles is largely elusive.

In the development and optimization of surfaces for virus removal, quantifying the reduction and understanding the mechanisms behind the reduction of infectious particles are crucial. Obtaining this information is often complex and time-consuming. Moreover, many of the used assays require expert knowledge outside the field of surface functionalization, for example, in microbiology. To quantify the infectivity of a sample that contains viruses, typically a combination of an end-point dilution and plaque assays is used.^5^ The outcome of these assays gives the number of infectious particles in the sample of interest. Easier quantification methods, which do however not asses infectivity, include counting intact viruses in flow virometry or scanning electron microscopy.^6,7^ These methods give the total number of virus particles per volume. With qPCR the copy number of genetic material of the virus of interest is determined.^8^ This copy number does not necessarily reflect the number of infectious virus particles present as virus inactivation may result in a release of genetic material. Both released genetic material and virus proteins do not contribute to infectivity; virus disassembly correlates with loss of infectivity. Current methods to monitor the breakdown of self-assembled complexes of bio(macro)molecules like the virus capsid include fluorescence correlation spectroscopy, fluorescence resonance energy transfer based methods, dynamic light scattering, nuclear magnetic resonance, and the monitoring of the presence of binding epitopes for antibodies only present in assembled viruses.^9−12^ The methods that are currently used to study virus inactivation and removal are experimentally complex and/or time-consuming and pose often a bottleneck in the development and optimization of antiviral surfaces.

Here we use single particle counting (SPC) in combination with easily accessible fluorescence microscopy and spectroscopy methods to investigate virus removal and inactivation. To showcase our approach, we functionalized a commercial PES microfiltration membrane with PEI as described in ref (4). Such membranes have been shown to effectively reduce the number of infectious virus particles; however, the details of the observed decrease in infectious particles are not well understood. We study virus removal and inactivation by these membranes using fluorescently labeled model cowpea chlorotic mottle viruses (CCMV). The SPC experiments are used to directly determine the fraction of intact viruses that pass the membrane. We subsequently use easily accessible changes in the emission spectra and fluorescence lifetime of the fluorescently labeled viruses to monitor virus disassembly and to determine the fraction of disassembled viruses.

For the simple, nonoptimized, membranes we used to showcase our approach, we determine a 2 log removal of intact viruses. The spectroscopy experiments, however, show that not only intact but also disassembled viruses are present in the filtrate. The intact and disassembled virus capsids found in the filtrate add up to ∼13% of the applied virus load. This implies that the majority of the viruses were captured by the membrane. We use fluorescence lifetime microscopy to image the virus particles adsorbed to the membrane. The fluorescence lifetime data show that most of the viruses adsorbed to the PEI-coated PES membrane are no longer intact but disassembled. Combined, the data show that the CCMV model virus not only adsorbs to the PEI coating on the membrane, the interaction with PEI results in the disassembly of the virus capsid.

## Materials and Methods

All chemicals were purchased from Sigma-Aldrich unless stated otherwise.

### Membrane Modification

For the membrane modification we followed the coating procedure reported in ref (13). In short, branched polyethylenimine (PEI, *M*_w_  ∼  25 kDa, Sigma-Aldrich), a cationic polymer, was adsorbed onto negatively charged commercial flat sheet EXPRESS Plus poly(ether sulfone) (PES) microfiltration membranes with a pore size of 0.45 μm (Merck Millipore, diameter 90 mm). The PES membranes were first cut into smaller pieces (diameter 20 mm) by using a punch and die set from Precision Brand. Before use, the stock PEI solution was diluted in demineralized water to 0.52 mM for coating of the PES membrane. Coating was achieved by overnight immersion of the membranes in a solution of 0.52 mM PEI under mild agitation. Afterward, the coated membranes were washed thoroughly with demineralized water, dried under ambient conditions, and stored until further use.

### Preparation of Fluorescently Labeled CCMV

Cowpea chlorotic mottle virus (CCMV) was obtained following the protocol reported in the literature.^14,15^ The solution exposed primary amines of the virus capsid proteins were targeted in a fluorescence labeling step. The formation of stable amide bonds between the amine groups on the capsid proteins and fluorophores was achieved by following the procedure reported in the literature.^16^ In short, the amine groups on the capsid proteins of CCMV were allowed to react with the *N*-hydroxysuccinimidyl (NHS) ester of Atto647N (ATTO-TEC GmbH). The labeling was performed by mixing a molar excess of the fluorophore into a CCMV solution (50 mM phosphate buffer, pH 7.5). This solution was incubated for 1 h at room temperature. Subsequently, the labeled viruses were separated from unreacted fluorophores by using a Zeba-spin desalting column (30 kDa molecular weight cutoff) and stored at 4 °C. Nonspecific binding of Atto647N to the virus particles was negligible.^17^

### Characterization of Fluorescently Labeled CCMV

The average number of attached fluorophore molecules per virus (degree of labeling or DOL) was derived from the absorbance at 260 and 646 nm. CCMV does not absorb at 646 nm; the molar extinction coefficient of the virus at 260 nm was reported to be 2.7 × 10^7^ M^–1^ cm^–1^ (5.87 cm^2^ mg^–1^).^18,19^ The molar extinction coefficient of Atto647N at 646 nm is 150000 M^–1^ cm^–1^; the absorbance of Atto647N at 260 nm is low with an extinction coefficient of 6000 M^–1^ cm^–1^. The fluorophore absorbance at 260 nm was taken into consideration in calculating the DOL. The labeling procedure resulted in a DOL of 61 fluorophores/virus.

### Filtration of Viruses

For the virus filtration experiments an Avanti mini extruder was used to support the (modified) membrane. The extruder was connected to 1 mL Hamiltonian syringes on both the inlet and outlet. With the help of a syringe pump (New Era & KD Scientific, Harvard, from Inacom Instruments), the flow of the virus solution through the membrane was set to a rate of 100 μL/min. All the filtration experiments were performed at room temperature.

### Quantification of Virus Concentration Using Single Particle Counting (SPC)

Virus particle counting experiments were performed as reported in ref (17). The virus quantification method is based on single particle counting of fluorescently labeled viruses using fluorescence microscopy. In short, fluorescence was excited by using a multimode 638 nm 2.1 W laser diode (Mitsubishi lasers, ML562G85-01) powered by a laser driver (Wavelength Electronics, LD5CHA-A). The emission was filtered by a band-pass filter from 650 to 710 nm (Chroma, D660/50) and an additional 647 nm long-pass filter (Semrock, blp01-647r) and imaged onto a camera (Basler, acA2440-75um). As sample substrates, we used #1.5 microscopy coverslips (Thermo Scientific) rinsed with spectroscopy grade ethanol (Ethanol Uvasol, Merck Millipore) and treated in a UV-ozone cleaner (Bioforce Nanosciences). On these microscopy coverslips FlexWell incubation chambers (Grace Bio-Labs) were placed to create wells. To individual wells, 50 μL of labeled virus solution was added. The wells were covered with another #1.5 microscopy coverslip to prevent evaporation of the solution. To quantify the number of virus particles in solution, we used an automated approach in which we located and counted diffraction limited spots in the images. We used the widely used Crocker–Grier algorithm implemented in the Python-based Trackpy package to locate and count the fluorescently labeled viruses.^20^ To exclude false positives, for example, from noise or (far) out of focus signal, an intensity threshold was used.

### Single Particle Tracking (SPT) to Determine Particle Sizes

For single particle tracking to obtain particle sizes, we used the same setup to recorded videos of the freely diffusing labeled virus particles in the solution. Excitation was synchronized to a 5 ms exposure time at a frame rate of 75 frames/s. To obtain particle trajectories, we used the same script as in SPC to identify particles in each frame. To avoid false positives, identified particles were required to be present for a least three consecutive frames. From the single particle trajectories mean-square displacements were obtained. The mean particle size was determined via the diffusion coefficient derived from the mean-square displacement data by using the Stokes–Einstein relation.

### Disassembly of CCMV in PEI Solutions

We incubated 200 μL of 0.52 mM PEI solution with 10 μL of a 1.6 × 10^12^/mL CCMV in an Eppendorf tube under agitation using an orbital shaker at 160 rpm for 30 min at room temperature.

### Spectroscopic Characterization

For the spectroscopic characterization of the labeled CCMV particles, we made use of several instruments. Emission spectra were recorded at λ_excitation_ = 630 nm by using a FluoroMax-4 (Horiba Jobin Yvon) spectrophotometer. Measurements of the fluorescence lifetime, fluorescence correlation spectroscopy (FCS), and 3D fluorescence lifetime imaging (FLIM) were done by using a PicoQuant MT200 confocal microscope equipped with a FlimBee laser scanner and a 60× NA1.2 objective (UPLSAPO60XW/1.20, Olympus). Atto647N was excited by a 640 nm pulsed laser; the emission was filtered with a 690/70 nm bandpass filter (AHF Analysetechnick, Germany) and detected on a single photon avalanche diode (PicoQuant, Germany). The obtained data were analyzed by using the SymphoTime64 software (PicoQuant, Germany).

## Results and Discussion

To mimic the presence of (enteric) viruses in the feed in a microfiltration experiment, a model nonenveloped virus, CCMV, was used. To enable quantification and to discriminate intact from disassembled viruses in fluorescence microscopy and spectroscopy experiments, the virus capsids were fluorescently labeled. We compare a PES and a PEI-coated PES microfiltration membrane to investigate the removal of infectious particles.

The concentration of a stock solution of fluorescently labeled viruses was determined spectroscopically. This solution was diluted, and the concentration of the diluted sample was determined by using single particle counting (SPC). Fluorescence microscopy videos of the individual, freely diffusing labeled CCMVs were recorded. SPC confirms the CCMV concentration to be 27 pM, as expected from dilution. Size determination by single particle tracking (SPT) of the labeled CCMV particles results in a mean diameter of 28 nm, which is in good agreement with the size expected for intact CCMV.^21^ The good agreement between the expected values and the measured concentration and size confirms that the solution mainly contains intact labeled viruses. This solution is used as feed in the filtration experiments.

**Figure 1:**
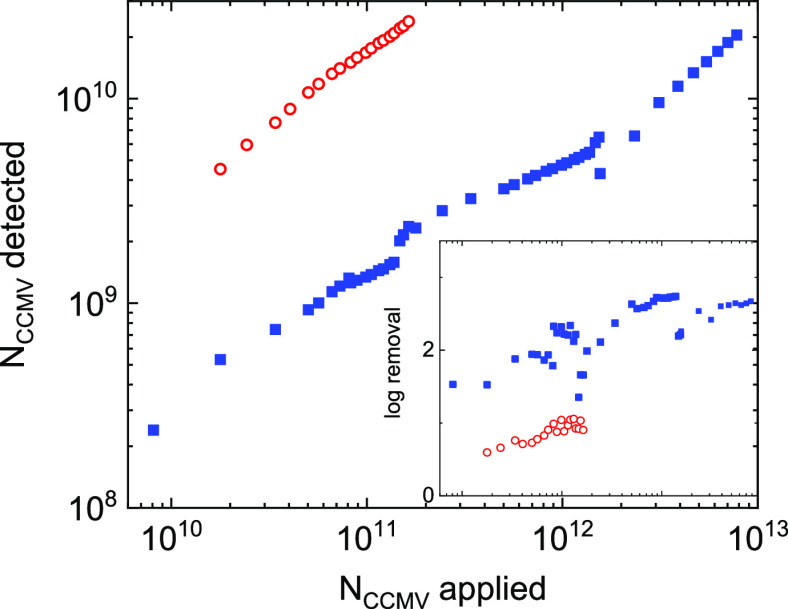
CCMV removal by microfiltration membranes. The amount of Atto647N-labeled CCMV particles was quantified in single particle counting experiments. The data show the cumulative amount of CCMV particles detected in the filtrate as a function of the cumulative amount of CCMV particles that was applied to the membrane via the feed. The cumulative CCMV removal by a PES membrane with a nominal pore size of 0.45 μm is shown in red, and the removal of a PEI-coated PES membrane is shown in blue. The inset shows the log removal as a function of the cumulative number of particles applied to the membrane via the feed.

In the first filtration experiment we compare the CCMV removal of a PES membrane with a nominal pore size of 0.45 μm to the CCMV removal of an identical PES membrane functionalized with PEI. The pore size of the PES membrane is known to be widely distributed; the pores are typically much larger than the size of single viruses. Size exclusion will therefore hardly contribute to virus removal. The 27 pM labeled virus solution was passed over the membrane, and aliquots of filtrate were collected in time. For each of the aliquots we directly determine the concentration of viruses using SPC. From the concentration we determined the total (cumulative) number of virus particles that was applied to the membrane and the total (cumulative) number that passed the membrane and is found in the filtrate. The cumulative number of applied and detected particles is presented in Figure 1. With increasing number of applied viruses (virus load) a proportional increase of the number of viruses was detected in the filtrate for both the bare and PEI-functionalized PES membranes. This shows that the virus removal stays constant for both membranes. Note that using SPC introduces an upper limit of particle concentrations that can be reliably determined. With increasing concentration the number of particles per frame becomes too large for accurate counting. This posed an upper limit of the concentration sampled. Over the virus load tested 70% of the viruses pass the PES membrane (0.5 log removal). For the PEI-coated PES membrane only 1% of the applied viruses is found in the filtrate (2 log removal) (Figure 1, inset). The PEI coating clearly results in a more efficient virus removal. These results are comparable to results obtained from plaque assays by using MS2 phages that were filtered over bare and PEI coated PES membranes. For MS2 phage a 1 log removal was observed for the bare PES membrane while PEI coating resulted in a 3 log removal.^13^ Increasing the CCMV load by 10 and 100 times gave the same 2 log removal; no sign of saturation of the membrane with CCMV was observed (Figure 1, inset). SPT experiments showed that the particles identified in the filtrate have a hydrodynamic radius of ∼28 nm. Fragments of the dissembled virus elude identification by SPT and SPC because compared to the intact labeled virus, the fluorescence of the labeled fragments is dim and the fragments diffuse fast. The presented log removal therefore refers to intact viruses.

**Figure 2:**
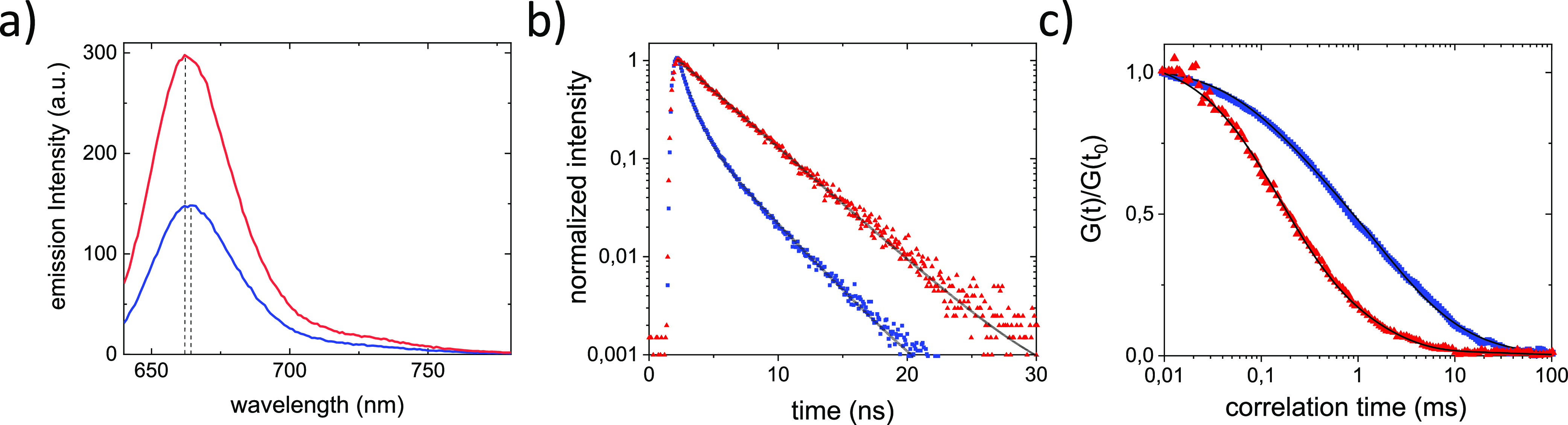
PEI-induced CCMV capsid disassembly in solution. (a) Emission spectra of Atto647N-labeled CCMV before (blue) and after (red) addition of PEI. The dashed vertical lines indicate the position of the emission peak maxima. (b) Peak normalized fluorescence decays of the CCMV particles before (blue) and after (red) addition of PEI. The lines through the data points represent a double- and single-exponential fit, respectively. (c) FCS autocorrelation curves of the Atto647N-labeled viruses in the absence (blue) and presence of PEI (red). The fit to the data is shown as a line. The data were normalized to *G*(*t*) at 0.01 ms.

To investigate if the earlier observed decrease in infective particles is not only an effect of the virus adhering to the microfiltration membrane, we investigate the effect of PEI in solution on CCMV integrity. For this purpose a high concentration of labeled CCMV was incubated with PEI in solution. To determine the effect of PEI on capsid integrity, fluorescence spectroscopy, fluorescence lifetime, and fluorescence correlation spectroscopy (FCS) measurements were performed. We previously reported that for labeled CCMV at the DOL used the fluorescence from intact virus capsids is quenched and red-shifted compared to the fluorescence from disassembled capsids.^17^ This difference in fluorescence can be used to study virus disassembly. In the bulk spectroscopy experiments on labeled viruses the presence of PEI resulted in dequenching of fluorescence and a blue-shift of the emission spectrum (Figure 2a). The peak fluorescence intensity doubled, and the peak position blue-shifted ∼3 nm in the presence of PEI (Figure 2a). In the lifetime measurements the addition of PEI caused the initial multiexponential fluorescence decay to convert to a single-exponential decay with a longer fluorescence lifetime of 3.7 ns (Figure 2b). This lifetime is close to that of the dye itself and represents noninteracting fluorophores on individual capsid proteins. FCS experiments showed that the addition of PEI resulted in a considerably faster diffusion of these particles (Figure 2c). The mean diffusion coefficient changed from ∼17 to ∼100 μm^2^/s. This change in the diffusion coefficient corresponds to the size expected for fully assembled and disassembled CCMV capsids. Together these experiments evidence that in solution the addition of PEI results in disassembly and hence inactivation of the CCMV.

**Figure 3:**
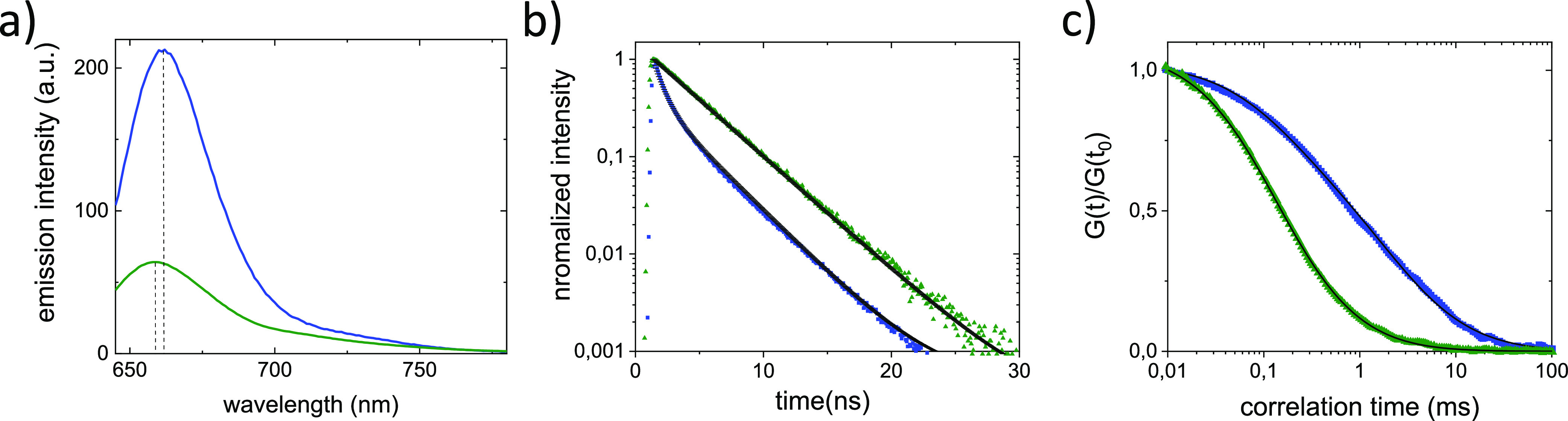
Disassembly of CCMV capsids upon filtration over a PEI-coated PES membrane. (a) Emission spectra of Atto647N-labeled CCMV of the feed (blue) and the filtrate (green). The dashed vertical lines indicate the position of the emission peak maxima. (b) Peak normalized fluorescence decays of the CCMV particles in the feed (blue) and in the filtrate (green). The lines through the data points represent a double- and single-exponential fit, respectively. (c) FCS autocorrelation curves of the Atto647N-labeled viruses in the feed (blue) and in the filtrate (green). The fit to the data is shown as a line. The data were normalized to *G*(*t*) at 0.01 ms.

Considering that incubation of CCMV with PEI results in virus disassembly, it is expected that filtration over the PEI-coated PES membrane not only results in virus retention but also affects virus integrity. To allow for the detection of disassembled virus capsids in the filtrate by using spectroscopic methods, an increased CCMV feed concentration of 270 pM was used. In Figure 3a, the bulk emission spectra of the feed and the filtrate are plotted. Compared to the feed, the peak emission intensity of the filtrate dropped by a factor of 4. This decrease in fluorescence is much less than expected based on the SPC experiments where 99% of the intact viruses were removed by filtration. The decrease in fluorescence intensity coincides with a blue-shift of the peak emission wavelength by 3 nm, which indicates the presence of disassembled virus capsids. The presence of disassembled virus capsids is further confirmed by fluorescence lifetime and FCS experiments (Figure 3b,c). The fluorescence decay of the filtrate can be approximated with a single exponential corresponding to a decay time of 3.7 ns. The FCS experiment gives a diffusion coefficient of ∼100 μm^2^/s. All these parameters confirm the presence of disassembled capsids in the filtrate. Concluding, both intact and disassembled virus capsids are present in the filtrate as evidenced by the single particle tracking and bulk spectroscopy experiments, respectively. A rough estimate of the ratio between assembled and disassembled capsids can be obtained from the spectra shown in Figures 2a and 3a. Disassembly of all virus capsids in the solution experiment resulted in an increase of the fluorescence by a factor 2 (Figure 2a). Considering that the filtrate only contains 1% intact capsids, the contribution of intact capsids to the total emission shown in Figure 3a can be neglected. We assume that all fluorescence observed in the filtrate originates from disassembled virus capsids. In the filtrate a decrease of the emission fluorescence intensity by a factor 4 is observed. Assuming that the virus capsids are either intact or fully disassembled, this means that 12.5% ((1/4)/2 = 1/8 = 12.5%) of the viruses that were present in the feed are present in the filtrate in the disassembled state. Determining the fraction of disassembled viruses is very difficult with other methods. A difference in the result from qPCR and a plaque assay, as observed for MS2 pages in microfiltration experiments, gives the difference between copy number of genetic material and the number of infectious particles.^8,13^ Because typically only a small fraction of viruses are infectious, this difference does not reflect the number of disassembled, inactivated viruses.

**Figure 4:**
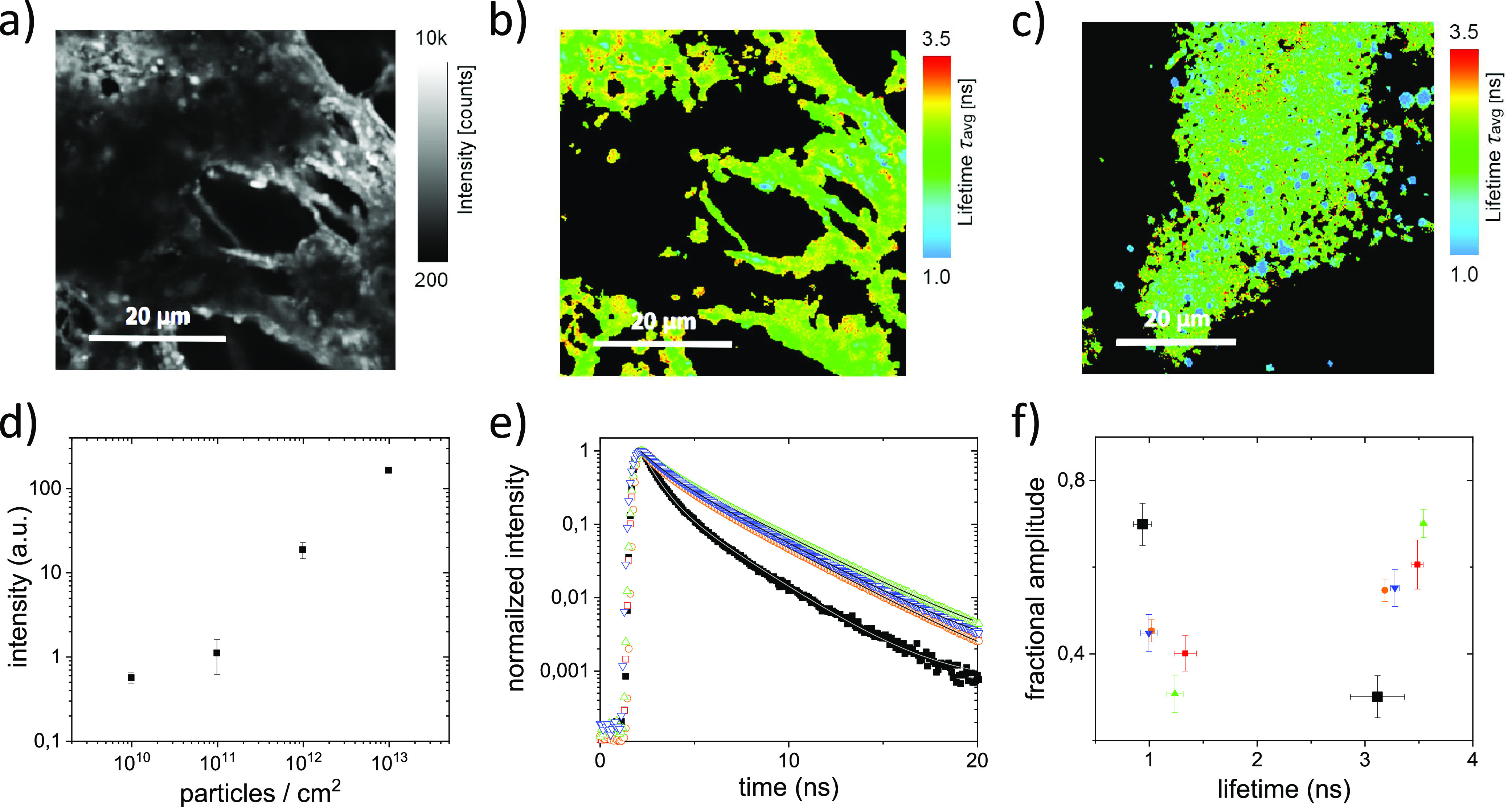
Fluorescence lifetime and intensity of labeled CCMV on PEI-coated PES membranes. (a) Fluorescence intensity measured on a PEI-coated membrane after exposure to a virus load of 7.7 × 10^12^ particles labeled with Atto647N. (b) Fluorescence lifetime image of the membrane shown in (a) obtained by fitting the measured fluorescence decays at each pixel to a single-exponential decay. (c) Fluorescence lifetime image of a PEI-coated PES membrane at a lower virus load of 7.7 × 10^11^ labeled particles. The fluorescence lifetime was obtained by fitting a single exponential to the fluorescence decay curves for each pixel. (d) Average fluorescence intensity per pixel on the PEI-coated PES membrane as a function of the virus load. The plotted intensity is corrected for differences in excitation power. (e) Peak normalized fluorescence decay observed on the filter after cumulative virus loads of approximately 10^13^ (red), 10^12^ (orange), 10^11^ (green), and 10^10^ virus particles/cm^2^ (blue) and the feed (black). The decay curves represent the average fluorescence decay over all virus containing pixels in an image. The gray lines represent double-exponential fits to the data. (f) Lifetimes and fractional amplitudes obtained from fitting a double exponential to average fluorescence decays shown in (e). The error bars show the standard deviation of the data obtained at different positions in the filter.

The capsids found in the filtrate only account for ∼13.5% (12.5% disassembled + 1% intact virus) of the total amount of virus applied to the filter. A large fraction of the viruses thus remained behind on the membrane filter. To confirm that viruses indeed remained behind on the membrane and to investigate whether these viruses are intact or disassembled, the membrane was inspected in confocal microscopy experiments. The fluorescently labeled viruses on PEI-coated PES membranes were imaged after exposure to different virus loads. The membrane was raster scanned, and time-correlated single photon counting (TCSPC) was used to record fluorescence decays and to obtain fluorescence intensity and FLIM images. Figure 4a shows a typical image of the fluorescence intensity at a cumulative virus load of 7.7 × 10^12^ particles. In the image, the large pore size of the membrane results in regions in which no fluorescence is observed. For lower loads qualitatively comparable images were obtained, but the total intensity per pixel decreased, reflecting the lower amount of virus on the membrane. Plotting the average intensity per pixel, considering the differences in excitation power used, shows an almost linear relation between the intensity and the applied virus load (Figure 4d). The intensity does not level off with increasing virus load, which implies that the membrane was not saturated with virus.

To establish if the viruses on the membrane are disassembled, a fluorescence lifetime image was obtained by fitting a single exponential to the fluorescence decay at each pixel (Figure 4b). Considering the limited number of photons detected per pixel, the analysis is limited to single-exponential fits and higher virus loads. For the lowest virus loads no meaningful fluorescence lifetime images were obtained. For virus loads of 7.7 × 10^12^ and 7.7 × 10^11^ particles the fluorescence lifetime images are shown in Figures 4b and 4c. Above (Figures 2b and 3b) it was shown that shorter lifetimes, resulting from interacting fluorophores, are associated with intact viruses. For disassembled viruses longer lifetimes are observed. On the membranes longer lifetimes dominate, but also shorter lifetimes can be found. At lower virus load a more patchy distribution of the lifetimes is observed.

To gain more quantitative insights, the fluorescence decays, obtained from all pixels in the images for each virus load, are summed. The resulting total decays are plotted together with the fluorescence decay observed for the intact viruses in the feed (Figure 4e). The fluorescence decay curves obtained on PEI-coated PES membranes overlap for all virus loads, including the ones obtained at low virus load. The fluorescence decay on the membrane, however, differs significantly from the decay observed for the intact labeled viruses in the feed. The fast decay that is associated with intact viruses is much reduced on the membrane. The total fluorescence decays are of high enough quality to fit a double-exponential decay. The fractional amplitudes and lifetimes obtained from the double-exponential fits of the data (Figure 4e) are presented in Figure 4f. For all samples decay components with lifetimes of approximately τ_1_ = 1.1 ns and τ_2_ = 3.4 ns are found. However, the fractional amplitudes of the decay components shift from *A*_1_ = 0.7 and *A*_2_ = 0.3 in solution to *A*_1_ ∼ 0.4 and *A*_2_ ∼ 0.6 on the membrane. The increase in the contribution of the longer fluorescence lifetime on the membrane evidences that viruses disassemble on the PEI-coated PES membranes. However, the fluorescence decay does not converge to the single-exponential decay observed for fully disassembled viruses (Figure 2b). The fractional amplitude of the shorter decay component indicates that ∼40% of the fluorophores still interact as they do on intact viruses. Note that this does not mean that the viruses are still intact. Adsorption to the membrane may cause the fluorophores to remain in close proximity although the viruses are no longer intact.

**Figure 5:**
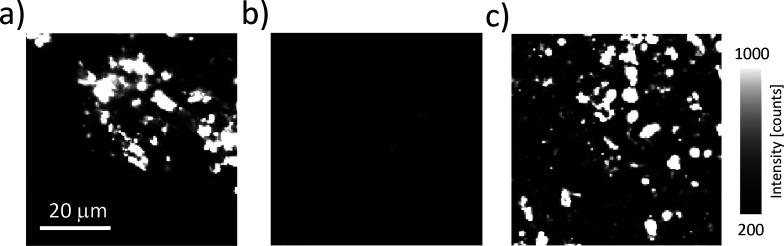
Fluorescence intensity of labeled CCMV on PEI-coated PES membranes: (a) before exposure to pH 3.8 and (b) after exposure to pH 3.8. (c) Adsorption of labeled CCMV at neutral pH after exposure to pH 3.8.

The adsorption and disassembly of the viruses on the membrane likely result from interactions between the positively charged PEI and the net negatively charged CMMV surface. To verify that adsorption is indeed dominated by charge–charge interaction, we changed the pH. A decrease in the pH should result in protonation of the CCMV surface while PEI remains protonated and the surface potential of the PES remains negative.^22^ Upon decreasing the pH, we thus expect the PEI to remain adsorbed to the PES membrane while the interaction strength between the PEI and the virus particles decreases. Indeed, we find that rinsing the virus containing membrane at pH 3.8 results in virus detachment as evidenced by the absence of fluorescent signal from the membrane (Figure 5a,b). Exposure of the pH 3.8 treated PEI-coated PES membrane to fluorescently CCMV at neutral pH results again in adsorption of CCMV. These observations confirm the electrostatic nature of the attraction between the virus and the PEI coating, and this opens up the possibility for membrane regeneration.

## Conclusion

Summarizing, we directly quantified the number of assembled virus particles in the filtrate and thus the log removal of the membrane. Additionally, our method allowed for the semiquantification of the fraction of disassembled, inactivated viruses in the filtrate, and the visualization of the inactivated viruses on the functionalized microfiltration membrane. Our data show that the interactions between PEI and the virus capsids is not only strong enough to hold back viruses on functionalized microfiltration membranes but that the interaction with PEI also results in the disassembly of the capsids and thus inactivation of the viruses.
